# Using dynamic time warping self-organizing maps to characterize diurnal patterns in environmental exposures

**DOI:** 10.1038/s41598-021-03515-1

**Published:** 2021-12-15

**Authors:** Kenan Li, Katherine Sward, Huiyu Deng, John Morrison, Rima Habre, Meredith Franklin, Yao-Yi Chiang, Jose Luis Ambite, John P. Wilson, Sandrah P. Eckel

**Affiliations:** 1 Spatial Sciences Institute, University of Southern California, Los Angeles, USA; 2 Department of Biomedical Informatics, University of Utah, Salt Lake City, USA; 3 City of Hope National Medical Center, Duarte, USA; 4 Department of Population and Public Health Sciences, University of Southern California, Los Angeles, USA; 5 Department of Computer Science and Engineering, University of Minnesota, Minneapolis, USA; 6 Department of Computer Science, University of Southern California, Los Angeles, USA; 7 Department of Civil and Environmental Engineering, University of Southern California, Los Angeles, USA; 8 School of Architecture, University of Southern California, Los Angeles, USA; 9 Department of Sociology, University of Southern California, Los Angeles, USA

**Keywords:** Asthma, Preventive medicine, Data mining, Data processing

## Abstract

Advances in measurement technology are producing increasingly time-resolved environmental exposure data. We aim to gain new insights into exposures and their potential health impacts by moving beyond simple summary statistics (e.g., means, maxima) to characterize more detailed features of high-frequency time series data. This study proposes a novel variant of the Self-Organizing Map (SOM) algorithm called Dynamic Time Warping Self-Organizing Map (DTW-SOM) for unsupervised pattern discovery in time series. This algorithm uses DTW, a similarity measure that optimally aligns interior patterns of sequential data, both as the similarity measure and training guide of the neural network. We applied DTW-SOM to a panel study monitoring indoor and outdoor residential temperature and particulate matter air pollution (PM_2.5_) for 10 patients with asthma from 7 households near Salt Lake City, UT; the patients were followed for up to 373 days each. Compared to previous SOM algorithms using timestamp alignment on time series data, the DTW-SOM algorithm produced fewer quantization errors and more detailed diurnal patterns. DTW-SOM identified the expected typical diurnal patterns in outdoor temperature which varied by season, as well diurnal patterns in PM_2.5_ which may be related to daily asthma outcomes. In summary, DTW-SOM is an innovative feature engineering method that can be applied to highly time-resolved environmental exposures assessed by sensors to identify typical diurnal (or hourly or monthly) patterns and provide new insights into the health effects of environmental exposures.

## Introduction

A standard approach in air pollution health effects studies is to relate continuously varying ambient exposures to health outcomes using exposure history summaries such as 24-h averages^1,2^. Current daily air quality regulations in the United States (US) are based on the Environmental Protection Agency’s (EPA) Federal Reference Method (FRM), which collects 24-h integrated samples and the Federal Equivalence Method (FEM), which collects hourly samples at Air Quality System (AQS) network monitoring sites. The EPA releases hourly, daily, and annual data, where the daily and annual summaries are sometimes averages of shorter-term measurements. By using averages, we may miss key short-term temporal variability in exposure that affects health differently than long-term averages. For example, Delfino et al.^3^ examined the impacts of air pollution “peaks” in a study of children with asthma and found stronger evidence for an association of asthma symptoms with the daily 1 h maximum of outdoor PM_10_ but not for the 24-h PM_10_ average. Personal and stationary air pollution monitors and low-cost air sensors provide highly time-resolved exposure data. New approaches are needed to summarize these data and explore potential health impacts.

Here, we focus on a new method to identify diurnal patterns in exposure time series data, especially under circumstances with slight time warping or shifting. For example, weekday NO and NO_2_ have a typical diurnal pattern with two periods of elevated levels related to the morning and evening traffic peaks. The morning peak of NO_2_ normally appears 1–2 h after the NO peak^4^. However, due to day-to-day variation in meteorology or traffic patterns (e.g., accidents), the weekday traffic peaks of a given pollutant (and lags between pollutant peaks) may not always occur at precisely the same time. We propose a new method that can identify typical diurnal patterns in exposure time series under temporal non-stationarity. Our method accounts for variations in the timing of the diurnal patterns by comparing segments from different time periods when comparing two daily time series.

Two broad approaches can be taken to discover typical patterns in time series data: (1) *supervised* time series classification when a priori grouping information (e.g., health responses) is available or (2) *unsupervised* time series clustering. Supervised time series classification targets patterns of exposure, for example, to discriminate between days where study participants did or did not have an asthma exacerbation. Unsupervised time series clustering characterizes observed exposure patterns independently of their association with health outcomes and can be used to address topics such as the diurnal patterns observed in daily indoor or ambient pollution exposures or how frequently these patterns occur. Patterns identified in an unsupervised clustering analysis can be later included as exposures in health models.

Time series clustering has been studied extensively over the past two decades, as summarized in a recent review by Aghabozorgi et al.^5^. Approaches used for time series clustering require a method for assessing the similarity between time series. Similarity metrics can be conceptualized as mathematical expressions that indicate the cost of transforming one time series into another or the inverse of the distance between two time series^6^. Simple Euclidean distance is one of the most widely applied similarity metrics. However, by definition, its elementwise alignment means it is unable to capture the similarity of shapes with small distortions in the time axis. Diurnal patterns in air pollution—which may impact human health—arise from complex processes, so it is important to anticipate small distortions over time (e.g., due to day-to-day variation in meteorology). Dynamic Time Warping (DTW) allows for elastic shifting of the time axis to detect similar shapes with different phases^7^, and many temporal proximity-based clustering methods use DTW as a similarity measurement^8–10^.

A Self-Organizing Map (SOM) is a clustering algorithm^11^ frequently applied in the exploratory phase of data mining. SOM transforms the input space onto a lower-dimensional (typically two-dimensional) gridded space to visualize and explore the properties of the input data. The standard SOM algorithm uses Euclidean distance as a similarity metric. Since Euclidean distance is ill-suited to characterize misaligned sequential data, several studies have refined the SOM algorithm by incorporating DTW^12–15^. However, these previous studies only replaced Euclidean distance with DTW in the matching phase of the algorithm but retained Euclidean element-wise alignment in the training phase of the algorithm that produces weights representative of a typical diurnal pattern. As a result, these previously proposed modifications to SOM are incomplete in their treatment of similar but misaligned patterns in sequential data and may produce suboptimal results.

In this paper, we propose a new Dynamic-Time-Warping Self-Organizing Map (DTW-SOM) algorithm that uses DTW as the similarity measure in both the matching and training phases of SOM. Thus DTW-SOM has the ability to better match similar patterns in time series with temporal misalignment as well as the potential to better characterize typical diurnal patterns. This novel methodological work was inspired by an application in environmental epidemiology, and we apply DTW-SOM to identify diurnal patterns in the residential particulate matter and temperature exposures of patients with asthma.

## Methods

### Dynamic time warping

**Figure 1:**
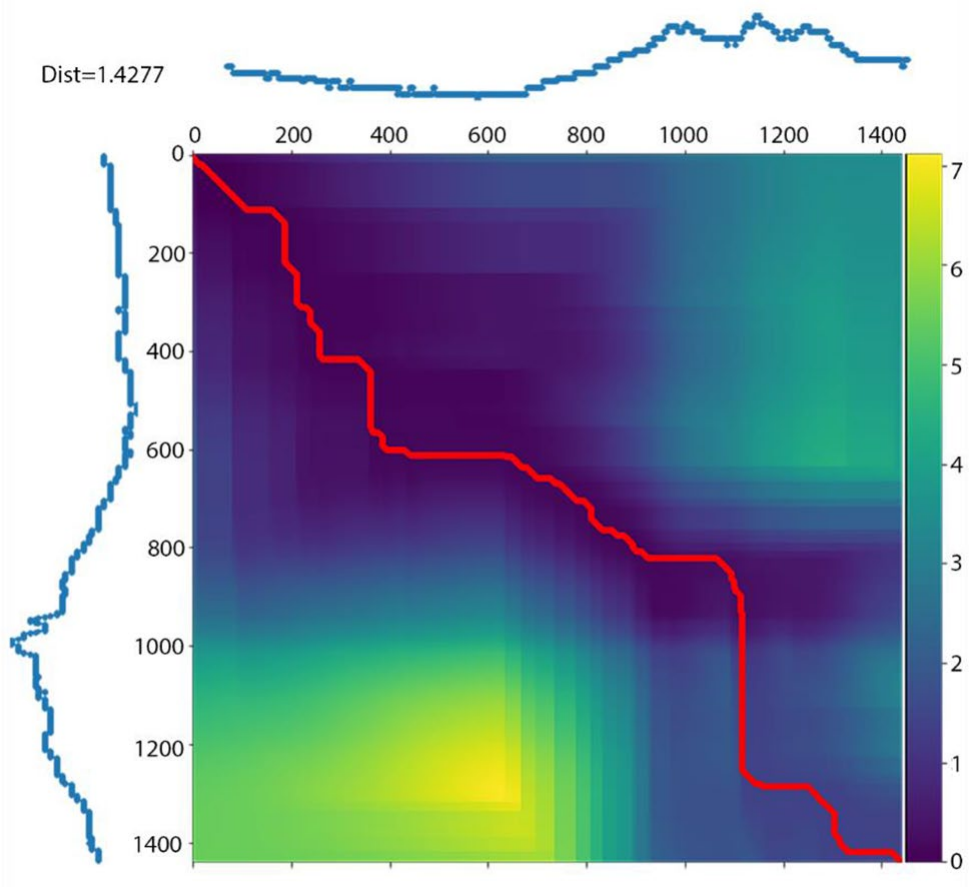
Dynamic Time Warping (DTW) alignment of two 24-h time series of outdoor temperature (at minute-level resolution so 60 × 24 = 1440 min long) from two measurement sites, with the color gradient displaying all pairwise Euclidean distances between time points (blue indicates the shortest distances) and the red line shows the shortest path.

DTW detects and matches the internal patterns of two time series of the same size by calculating a two-dimensional distance matrix with all possible pairwise Euclidean distances between time points (Fig. 1). DTW alignment is determined by finding the shortest path (i.e., the red line in Fig. 1) that minimizes the overall combined values of the distance matrix under: (1) a boundary condition by which the path starts from the top-left and ends at the bottom-right corner to ensure that the alignment does not partially cover subsequences; (2) a monotonicity restriction which requires that the path cannot go back in time to ensure that the internal patterns will not be repeatedly used in alignment; and (3) a continuity restriction that does not allow the path to break in time to ensure that no internal patterns are omitted. The DTW distance is then calculated by summing the Euclidean distance values along the shortest path. DTW can still be applied in cases with missing data because the matrix does not have to be square.

DTW is computationally intensive with a quadratic time and space complexity, O(N^2^), that limits its use with large time series. However, many optimization techniques such as lower bounding, early abandoning, run-length encoding, bounded approximation and hardware optimization have been used to develop more efficient versions of DTW^16^. We initially considered fast-DTW^17^, which is an approximation of DTW that has a linear time and space complexity, O(N). Fast-DTW relies on three key operations. First it “coarsens” the data into smaller time series with coarsened time resolution. Second, it finds the minimum-distance warp path at the coarser resolution. Third, it refines the warp path through local adjustments at finer resolutions. However, recent research claimed that fast-DTW is generally slower than the exact DTW in realistic data mining applications^18^. Constrained-DTW is another optimization technique which narrows the search window around the diagonal of the warping matrix using global constraints. Different types of constrained-DTW have differently shaped search windows. Two frequently used global constraints are the Sakoe Chiba band^19^ and the Itakura parallelogram^20^. In this study, we compared two optimization techniques against the standard DTW, which are fast-DTW and constrained-DTW with the Sakoe-Chiba band, to develop the best DTW-SOM implementation.

### Self-organizing maps

SOM is a type of unsupervised neural network with an input layer and an output or mapping layer. The neurons in the input layer and the mapping layer are fully connected, which means that each neuron in the input layer is connected to each of the mapping neurons and vice versa. Each weight on a connection in SOM represents the similarity between the connected mapping neuron and the input neuron. At each iteration of training, SOM searches for the mapping neuron whose weights are most like the input data (input vectors). The neuron with the best match is called the Best Matching Unit (BMU). This training regime is called competitive learning as opposed to the error-correction learning strategy used in other standard neural networks.

**Figure 2:**
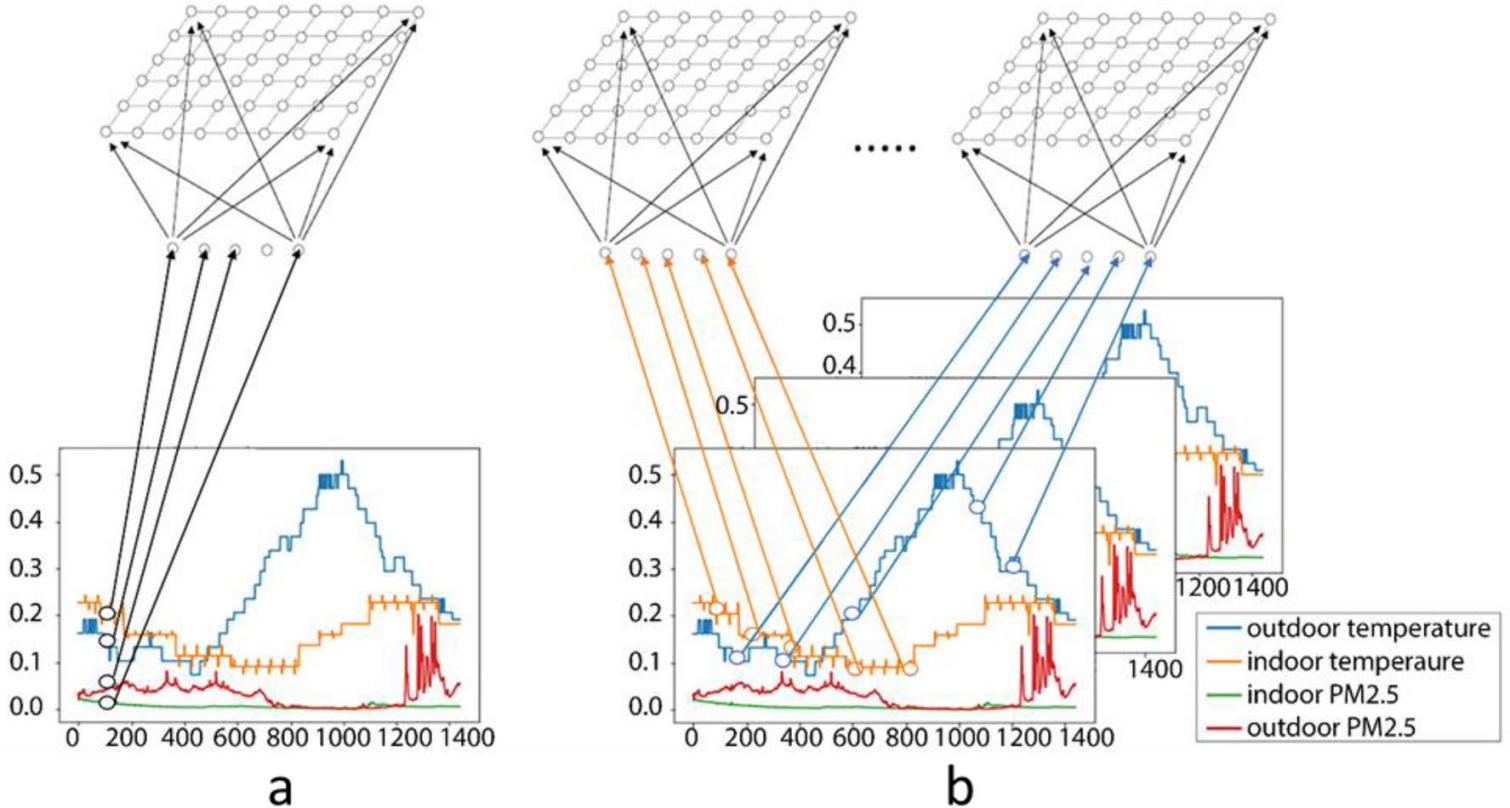
Conceptual diagrams showing the use of SOM to: (**a**) discover multipollutant patterns, as in Pierce et al. (2014); and (**b**) diurnal patterns of a single pollutant, as proposed in this study.

SOM has been widely applied in time series clustering^21^. For example, SOM was used in an air pollution epidemiology study to classify daily levels of several particulate and gaseous air pollutants into a set of multipollutant profiles, effectively identifying groups of days with similar daily average pollutant mixture patterns^22^. This study sought to identify pollutant mixture co-occurrence patterns using daily averages of 10 ambient air pollutants acquired from a US EPA Air Quality System (AQS) monitoring station in Atlanta from 2000 to 2007. For the current study, we sought to use SOM to discover the typical *diurnal* patterns for a single exposure (e.g., PM_2.5_) using PM_2.5_ concentrations at each minute of a day from 7 sites/homes and 1 year. Figure 2 shows the conceptual differences between the deployment of SOM in the two studies.

### Dynamic TIME WARPING SELF-ORGANIZING MAPS

**Figure 3:**
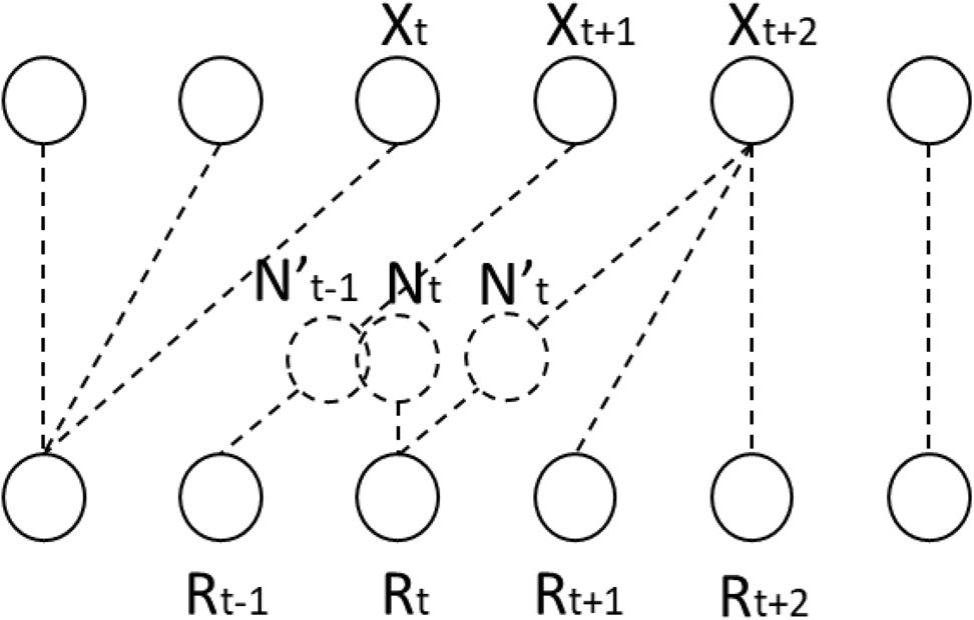
Calculation of new neuron weights *N*_*t*_ using DTW alignment. *N’*_*t*_ and *N’*_*t-1*_ are the weighted means of two adjacent DTW pairs; however, neither match existing timestamps. To calculate *N*_*t*_, we approximate its value using *N’*_*t*_ and *N’*_*t-1*_*.*

Our proposed DTW-SOM has two key differences from the standard SOM. First, inspired by previous studies^12–15^, we replaced the Euclidean distance similarity measure with DTW distance so that: (a) the BMU of a data sample is defined as the neuron in the output space with the minimal DTW distance to the data sample; (b) the distance-decay kernel function uses DTW distance; and (c) the weights of the neighborhood neurons for the BMU are updated under the kernel function using DTW distance. Second and most importantly, we developed a novel training regime for DTW-SOM. In the standard SOM, for a neuron *r*, the rule for updating weights *W*_*r*_ corresponding to input *x* is given by:

1 $${W}_{r}^{new}={W}_{r}^{old}+\varepsilon \bullet {h}_{rs}\bullet \left(x-{W}_{r}^{old}\right)$$

where $\varepsilon$ is the learning rate, *h*_*rs*_ is a distance-decay kernel function between neuron *r* and BMU *s*, and as the distance between *r* and *s* declines, *h*_*rs*_ increases. When *r* equals *s*, *h*_*rs*_ is at its largest. From Eq. (1), we can see that the new weights are weighted means of the old weights and the input *x* under Euclidean alignment. However, if the BMU *s* is determined by DTW distance, this updating rule will adjust the weights of *r* towards an input *x* using Euclidean alignment, which is very likely not the one with the minimal Euclidean distance. To solve this issue, we revised Eq. (1) to update weights *W*_*r*_ using DTW alignment. For any *W*_*rt*_ which is the value of *W*_*r*_ at timestamp t:

2 $${W}_{r\tilde{t }}^{new}={W}_{rt}^{old}+\varepsilon \bullet {h}_{rs}\bullet \left({x}_{\overline{t} }-{W}_{rt}^{old}\right)$$

where ${x}_{\overline{t} }$ is the value of x at the timestamp that is aligned to *W*_*rt*_ by DTW, and $\tilde{t }$ is a new timestamp between *t* and $\overline{t }$. One major challenge in the revision is that the DTW alignment is not always one-on-one. Sometimes one *W*_*rt*_ will be aligned to more-than-one ${x}_{\overline{t} }$, or vice versa. Moreover, under the elastic DTW alignment, ${W}_{rt}^{new}$ is calculated by the value at $\overline{t }$ in *x* and the value at *t* in *W*_*r*_, so it would best represent the new weights at a timestamp $\tilde{t }$ between $\overline{t }$ and *t*. As a result, not all the new time stamps $\tilde{t }$ match existing timestamps (Fig. 3). To calculate the new weights at each existing timestamp, we used the pseudo code in Table 1 to approximate its value with all of the adjacent new values generated by Eq. (2).

**Table 1:**
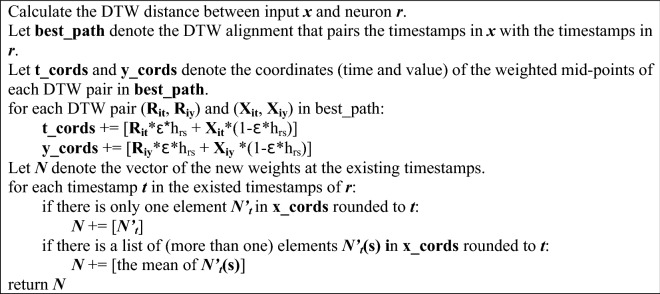
Pseudo code of DTW-SOM updating rules.

### Data description and preprocessing

The Pediatric Research Using Integrated Sensor Monitoring Systems (PRISMS) Utah Informatics Platform Center conducted a panel study of 10 participants with asthma (ages 5–51, 4 children and 6 adults) in 7 households near Salt Lake City, UT from April 2017 to April 2018. These 7 households included a total of 16 children ranging from younger than school age to late teens, some of whom participated in the study. In 2 households, a non-participant child was later diagnosed with asthma. Residential indoor and outdoor PM_2.5_ and temperature were measured using two sensors located inside and one located outside the home. The deployed sensors were a commercial Dylos Corporation particle counter, modified to include sensors for relative humidity and temperature, and Wifi communications. The conversion of particle counts to μg/m^3^ follows rules suggested previously^23^. Relative humidity is used to calibrate the raw PM_2.5_ readings and was not of primary interest, so we did not include it in our subsequent analysis. Participants (or their guardians, for some child participants) were asked to submit daily questionnaires about asthma symptoms and medication use in the past 24 h, including frequency of use of rescue medication (“How often did your child use an Albuterol or Xopenex inhaler or receive a nebulized treatment in the last 24 h?”). Daily questionnaires were submitted electronically before bedtime (typically ~ 8 p.m.). All the data was collected with informed consent form either the participants (for adults) or their parents or legal guardians (for under 18 children).

We applied several data processing steps. To start, we used a median filter with a kernel size of 3 (the minimum size required to remove the extreme outliers in our test runs) to smooth the signals. In some instances, more than one sensor was collecting data in a given microenvironment (either indoors or outdoors). To reduce the time series from the two sensors into a single time series, we calculated the means of the minute-level concentrations. Future work might consider alternative methods (e.g., choosing the maximum or using a single representative sensor). The time series contained missing data for various reasons (e.g., sensors stopped working, malfunctions in the data transfer pipeline). We first truncated the data to remove time periods with consecutive missing values longer than 1 h, and we then used a bidirectional linear interpolation method to fill in periods of missing values < 1 h in duration. Next, we used a min–max scaler to rescale all pollutant values from 0 to 1. Then, we used a non-overlapping 1-day windowing approach to separate the data into daily time series starting from 8:00 p.m. and ending at 7:59 p.m. on the next day, to align with the typical questionnaire response time. Finally, we excluded incomplete days with < 1440 timestamps (the total number of minutes per day). Therefore, each daily observation has 4 time series each with length 1440: indoor temperature, outdoor temperature, indoor PM_2.5_, and outdoor PM_2.5_. Matching days with available exposure data to days with available daily asthma questionnaires resulted in a total of 823 days with complete data for both exposure time series and questionnaires from 10 patients in 7 households.

### Ethics approval

The methods and experimental protocols used for this study were reviewed and approved by the Institutional Review Boards at the University of Utah and University of Southern California. All methods were carried out in accordance with relevant guidelines and regulations.

### Informed consent

All the data was collected with informed consent from either the participants (for adults) or their parents or legal guardians (for children < 18 yrs).

## Results

### DTW-SOM implementation

When developing our implementation of DTW-SOM, we compared the running time of 3 DTW variants (standard DTW, fast-DTW, and constrained-DTW with the Sakoe-Chiba band) on 100 iterations using random choices from our data samples (i.e. 24 h of outdoor temperature data at a minute-level resolution with 60 × 24 = 1440 timestamps) under the same computer settings (i.e. Intel Core i9-8950HK CPU @ 2.90 GHZ, 64.0 GB RAM, × 64-based processor). We found that it took 0.0086 s on average (range: 0.0079 to 0.0113 s) to run constrained-DTW with a Sakoe-Chiba band of size 60, whereas it took 0.0115 s of running time on average (range: 0.0108 to 0.0132 s) for standard DTW. In contrast, fast-DTW surprisingly cost 0.6127 s of running time on average (range: 0.5061 to 0.7887 s) and was actually slower than standard DTW, thereby confirming the findings of Wu and Keogh^18^. In this paper, we used the Python implementation by Tavenard et al.^24^ for standard DTW and constrained-DTW. The standard DTW Python implementation by Meert et al.^25^ took 4.449 s on average (range: 4.250 to 4.752 s). We found standard DTW and constrained-DTW results to be < 1% different in terms of distances and have the same warping path in most of the test runs on our data samples. So, we applied constrained-DTW as a computationally efficient approximation to exact DTW in our DTW-SOM algorithm. When training DTW-SOM using our data, it generally took ~ 3000 updating iterations for the weights to converge. Within each iteration, the algorithm needs to calculate DTW paths and distances between the target neuron and its neighboring neurons (depending on the distance-decay functions) in hundreds of data samples (depending on the training batch size). So, the computational time savings from using constrained-DTW instead of standard DTW (0.0029 s on average) translates to hours of computational time savings when training the DTW-SOM.

### DTW-SOM vs. previous SOM algorithms

**Figure 4:**
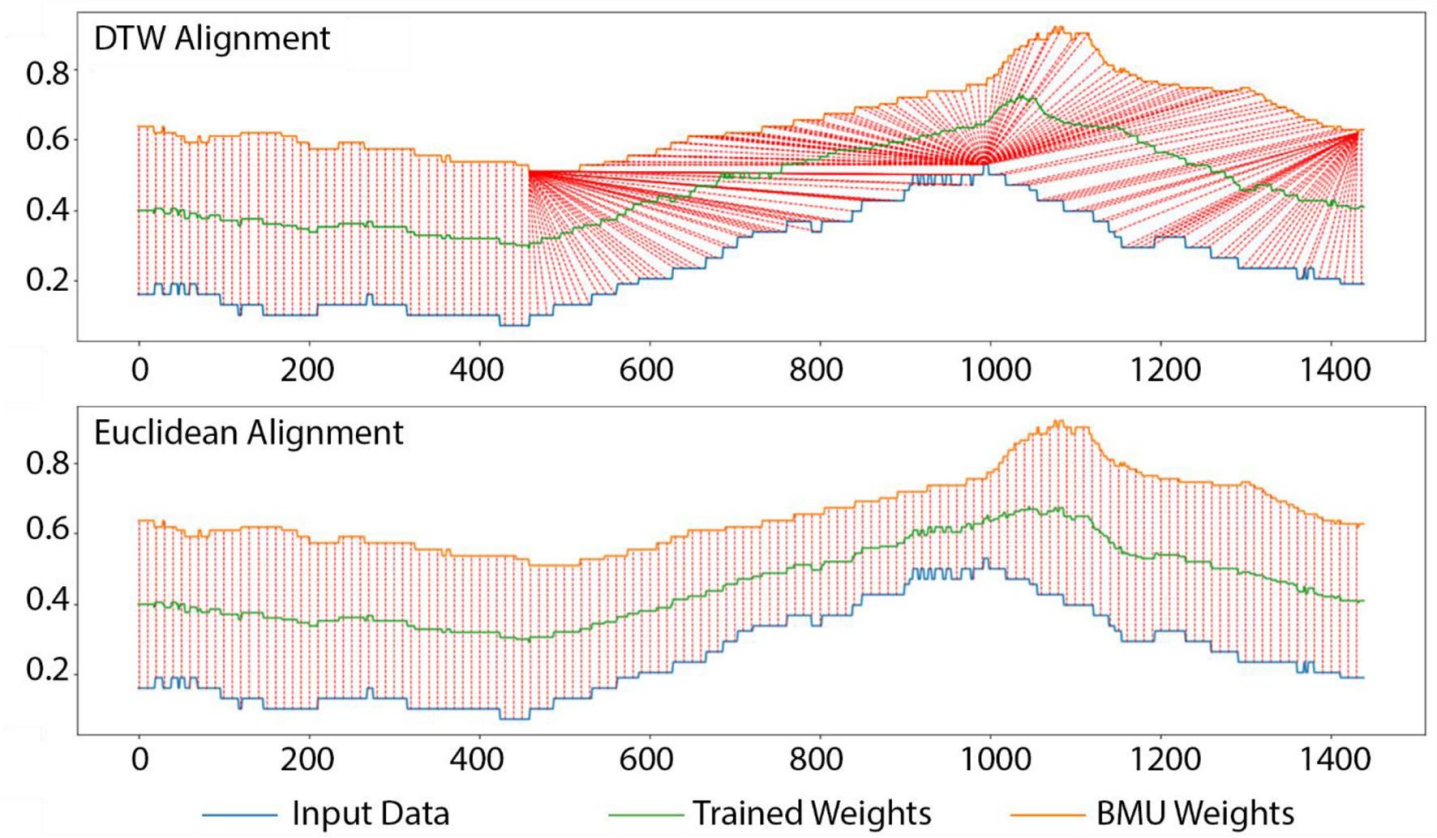
Visual comparison of the trained weights resulting from one iteration of the Euclidean training rule vs. the DTW training rule applied to a randomly selected observation from the 24-h outdoor temperature time series. Input data refers to the standard time series, trained weights refer to the weights after the iteration, and BMU weights refer to the weights at the previous iteration.

**Figure 5:**
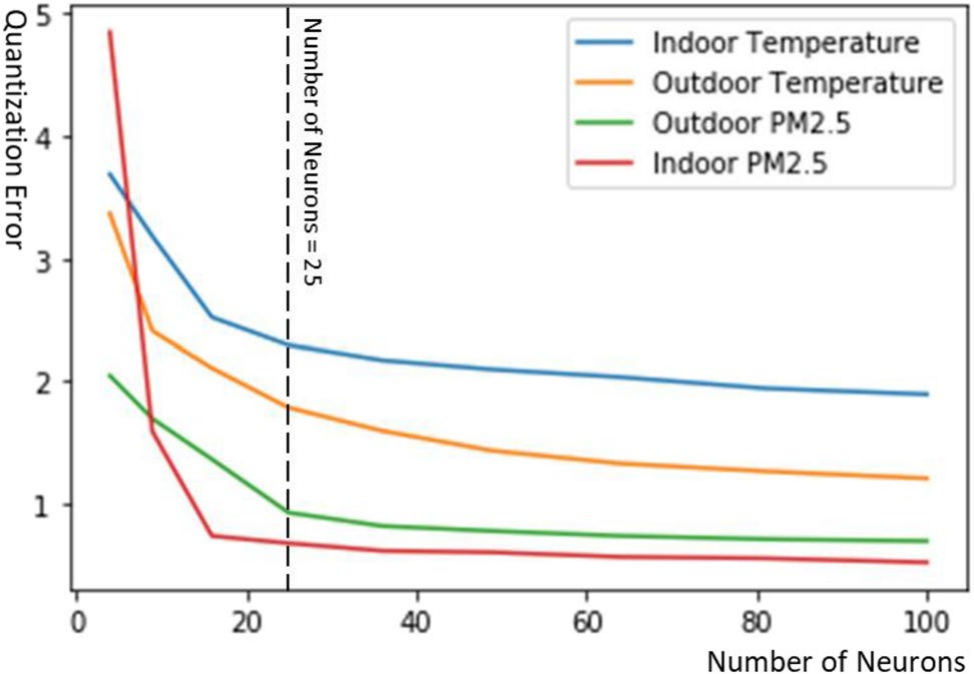
Quantization error as a function of the number of neurons in DTW-SOM.

We first demonstrate the difference between the alignment and training rules in standard SOM vs. DTW-SOM by reporting the intermediate results of a single iteration of each when applied to a single exemplar input data sample which was randomly selected from the 24-h outdoor temperature time series (Fig. 4). We sought to demonstrate the similarity between the trained and initial weights under the two training rules and to compare their ability to maintain the patterns of the input time series during training. Then, under the assumption of a fixed size output space (this parameter was tuned in Fig. 5), we compared quantization errors (the root-mean-square error of moving each input data to the centroid of its cluster) from: (a) standard SOM with Euclidean distance measurement; (b) SOM with only DTW distance replacement; and (c) DTW-SOM as proposed in this paper. Figure 4 compares the unique training rules of DTW-SOM using DTW alignment with the common training rules of previous SOM algorithms using standard timestamp-wise alignment, by displaying the one-minute resolution input time series of measured ambient temperature (blue line) and its BMU (orange line), and the intermediate training results of the updated BMU after one iteration (green line). The red dashed lines delineate the alignment for updating the weights of the BMU. In both subplots, the orange BMU was determined by DTW distances. The updated BMU retains the clear peak observed in the input data under the DTW training rules. However, the updated BMU appears “smoother”, having lost the peak in the input data, under the timestamp-wise alignment training. This plot only shows one training iteration. After many training iterations, the weights adjusted by the standard SOM rule will largely lose the peak pattern even though the BMU at each iteration was picked by DTW distances (this conclusion is supported by the following experimental results). We calculated and compared the Euclidean distances between the trained weights and the input data under both scenarios, as well as the variances of the trained weights, the initial weights, and the input data to quantify the variation in the representative pattern extracted by the two training rules (Table 2).

**Table 2:** Quantitative comparison of the trained weights resulting from one iteration of the timestamp training rule vs. the DTW training rule on a randomly selected observation from the 24-h outdoor temperature time series.

Summary statistic	Timestamp Alignment	DTW Alignment
Euclidean distance between the trained weight and the initial (BMU) weight	7.77	7.37
Variance of the trained weights^a^	0.012	0.014

^a^Variance of the input time series was 0.017 and the variance of the initial BMU weights was 0.010.

Table 2 shows that the trained weights are still closer to the input time series under the DTW training rules than under the timestamp training rules (even under Euclidean distance measurement), suggesting that the DTW trained weights better represent the input data. The variance of the weights from the DTW training (0.0137) was closer to that of the standard input time series (0.0164) than that of the Euclidean training (0.0118), indicating that DTW trained results better preserved the details of the input data.

To optimize the number of SOM output space neurons (clusters), we computed quantization errors for all 4 variables using DTW-SOM and different numbers of neurons. According to the inflection points of the 4 curves (Fig. 5), we chose a 5 × 5 output space with 25 neurons for comparing the quantization errors for all three SOM variants. DTW-SOM had the lowest quantization error for all 4 variables (Table 3). Moreover, to demonstrate that the temporal patterns extracted by DTW-SOM could better represent the input time series, we compared the final output neurons’ weights from DTW-SOM and standard SOM using outdoor temperature since the raw data exhibited a regular diurnal trend (i.e., peak temperature at midday). To examine whether the topological relationships in the input data were preserved by the SOM algorithms, we stratified the input observations by season and used bar plots for each neuron to show the seasonal distribution of input observations best matching the given neuron.

**Table 3:** Quantization errors from applying the three SOM algorithms, each with a 5 × 5 output space (25 neurons), separately to each of the four residential sensor readings.

	Outdoor temperature	Indoor temperature	Outdoor PM	Indoor PM
Standard SOM with Euclidean distance measurement	1.675	2.476	0.924	0.676
SOM with DTW distance measurement	1.706	2.404	0.909	0.717
DTW-SOM	1.189	1.800	0.907	0.538

**Figure 6:**
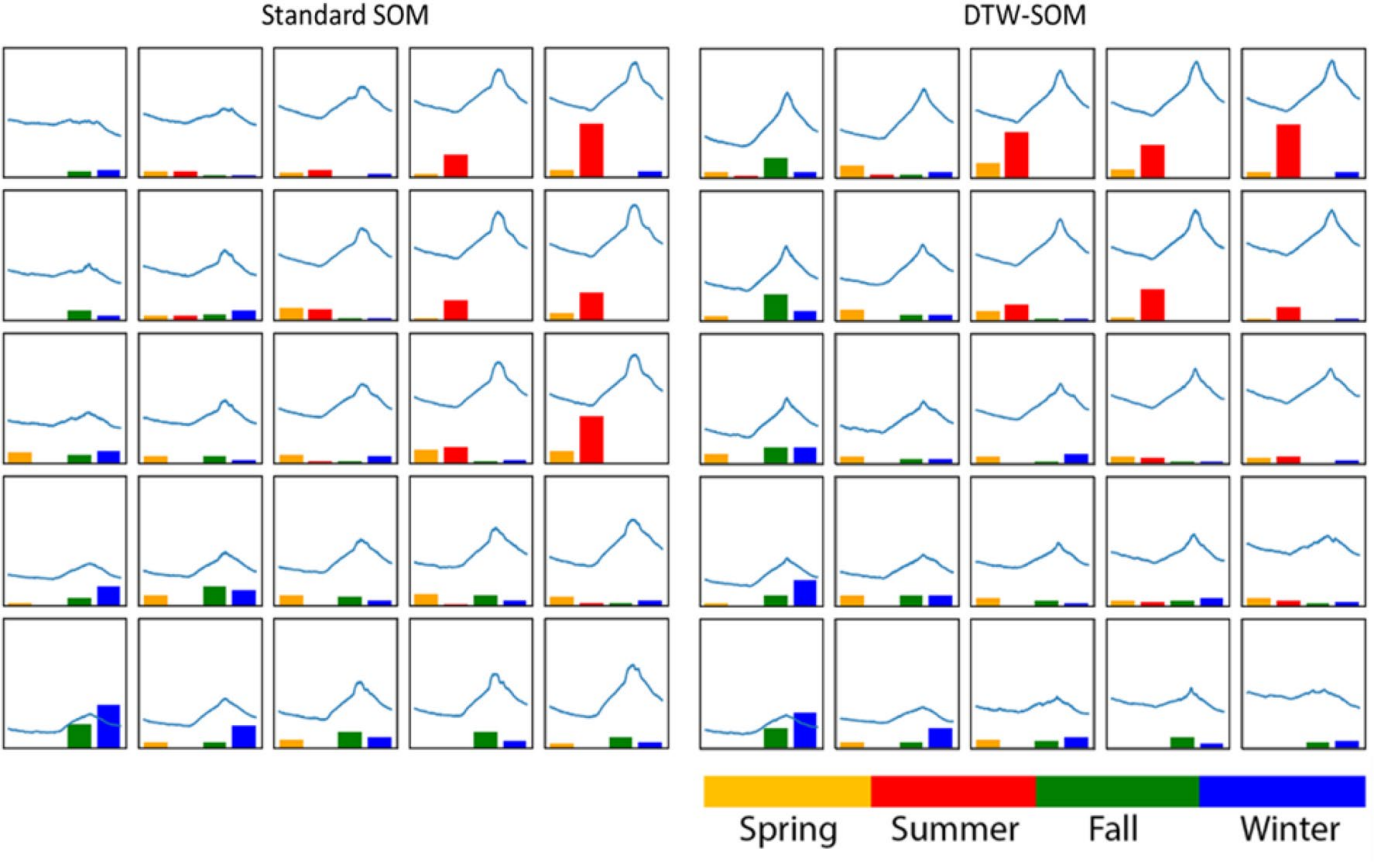
Diurnal patterns in outdoor temperature identified using standard SOM (left) and DTW-SOM (right). Each cell represents a neuron, the curved line represents its final weights, and the bar plot indicates the distribution by season of the input observations best matching that neuron.

In Fig. 6, both SOM methods produced a topological transformation of the diurnal patterns of outdoor temperature into the 2-D spatial relationship. For example, the cells in the upper right corner of both subplots display high temperature with noontime peaks, and the input observations that best match those cells are mostly summer days. However, several of the standard SOM neurons have “flattened” or “distorted” peaks due to the Euclidean updating rules. Moreover, the average Euclidean distance of each neuron from its eight Moore (nearest surrounding) neighbors was 0.51 for standard SOM and 0.47 for DTW-SOM. This indicates that the DTW-SOM produced stronger neighborhood relationships than the standard SOM in the 2-D output space. When stratifying by season, we found that the gradual changes in patterns across the output map corresponded well with the expected seasonal differences in temperature for both methods. For both subplots, observations in the lower left were mainly from winter and those in the upper right were mainly from summer. DTW-SOM produced a slightly more distinct separation of observations across different seasons, based on season-specific intra-cluster purity (DTW-SOM entropy: 3.27 and standard SOM entropy: 3.29). Based on these results, we conclude that DTW-SOM better transformed the internal relationships of the input time series into spatial relationships on the 2-D maps.

### Asthma inhaler usage and diurnal patterns in residential indoor and outdoor PM_2.5_

**Figure 7:**
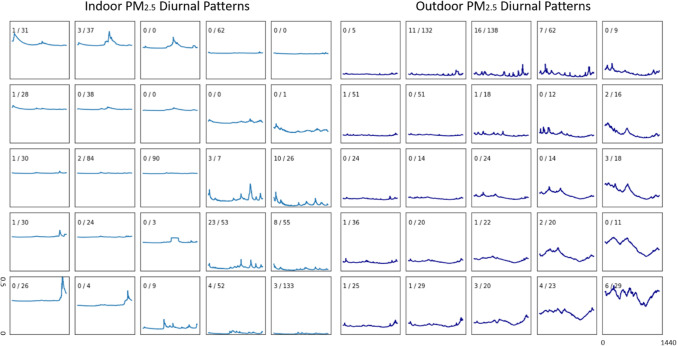
Diurnal patterns in indoor (left) and outdoor (right) residential PM_2.5_ identified using DTW-SOM. The fraction in each cell represents the number of days with inhaler usage over the number of days matching the diurnal pattern.

We used DTW-SOM to explore diurnal patterns in indoor and outdoor residential PM_2.5_ (Fig. 7). We overlaid counts of daily asthma inhaler use on the identified diurnal patterns. For indoor PM_2.5_, days with lower levels but more variation (lower right-hand corner) appeared to have more reports of inhaler use. For outdoor PM_2.5_, days with lower levels but more variation (top middle part) and days with sinusoid diurnal patterns (lower right-hand corner) tend to have more reports of inhaler use. This is a proof-of-concept demonstration, which could be formalized in future data analyses as outlined in the discussion.

## Discussion

In this article, we developed and introduced DTW-SOM, a new time series clustering method based on SOM, and used it to identify typical patterns in highly time-resolved air sensor data. We aimed to both illustrate the novelty of DTW-SOM and highlight the significance of time series pattern discovery using PM_2.5_ exposures as an example. The novel aspect of DTW-SOM as a pattern-based clustering method is that it uses dynamic time warping as the similarity measure in both the matching and training phases of SOM. Previous variants of SOM had incorporated DTW in the matching phase. Using data from four environmental exposures in our motivating application, we compared DTW-SOM to other SOM algorithms and found that DTW-SOM produced the lowest quantization errors in clustering and the highest purity within each output neuron (evaluated by entropy). DTW-SOM also preserved more details of the input time series (evaluated by variance), better preserved the topology relationship of the input data and better summarized time series patterns (e.g., retained diurnal temperature peaks).

DTW-SOM was designed for pattern discovery in highly time-resolved time series data and theoretically could be applied to many realms where there is a need to analyze patterns in temporal or serial data such as signal processing, biology, aerospace, finance, medicine, and meteorology^26^. Using data from our motivating application, we used DTW-SOM to cluster daily outdoor temperature time series and discovered the expected typical diurnal patterns, which varied by season. We also applied DTW-SOM to indoor and outdoor PM_2.5_. Indoor residential PM_2.5_ has considerable variation day-to-day as well as variation across households due to differences in the residences themselves, the resident’s habits, and the indoor sources. This variation makes it challenging to identify a typical diurnal pattern for indoor PM_2.5_. These patterns likely vary by household. The dynamic time-warping component of DTW-SOM helped to minimize issues related to, for example, the preparation of dinner (and the accompanying combustion-related spikes in indoor PM_2.5_) at slightly different times each day. Conceptually, outdoor PM_2.5_ should have more identifiable diurnal patterns. All of these issues should be carefully considered in future work. Future studies incorporating diurnal patterns in exposure identified through DTW-SOM will likely want to perform dimension reduction to identify the key diurnal patterns related to health, while accounting for key confounding variables. Typical diurnal patterns offer a complementary approach to summarizing participants’ exposure history (vs. the typical 24-h, weekly, or monthly averages) and take advantage of the novel information provided by sensors measuring environmental exposures. These novel summaries may provide new insights into exposure patterns, as well as associations between these exposure patterns and selected health outcomes. We applied DTW-SOM to daily time series, but DTW-SOM could be applied at shorter or longer time scales as well, such as hourly or monthly.

A methodological limitation of DTW-SOM as presented here is that it supports only univariate time series. Future extensions could include a multivariate version of DTW-SOM. In comparison with standard SOM, DTW-SOM required substantial computation time, even though this has been largely mitigated by choosing an optimized DTW implementation. Future research on novel DTW optimization techniques could advance DTW-SOM in more data-extensive situations. Moreover, DTW as a shape-based similarity measurement has been found to have limitations in a few situations (i.e., phase differences between two time series)^26^, thus investigations on novel DTW variants, for example weighted DTW (WDTW) or derivative DTW (DDTW), may support the use of DTW-SOM with more complex time series data. At last, DTW-SOM is an unsupervised learning method and it may be worth investigating supervised methods for identifying clusters of exposure time series most related to health outcomes.

## Conclusions

The clustering of time-series data to extract valuable information (e.g., patterns) from complex and massive datasets is a major focus in many scientific domains. SOM is one of the most popular unsupervised approaches. Aghabozorgi et al.^5^ classified the purpose of time series clustering methods into three categories: (1) recognizing dynamic changes; (2) prediction and recommendation; and (3) pattern discovery. However, the reliance on conventional Euclidean similarity measurement in the standard SOM, pattern discovery objective has not been adequately addressed. DTW-SOM provides a new framework for pattern-based feature engineering of time series, such as those produced by the growing number of sensors used in studies of human health. This paper shows how the resultant clusters of exposure time series patterns offer a complementary method for summarizing exposure histories beyond the simple summary statistics commonly used in health studies. For example, we see in Fig. 7 an indoor PM_2.5_ pattern with high variations and peaks in the late afternoon and early evening that was associated with a high rate of days with asthma patients using inhalers (23/53). Such a pattern would be hard to depict using summary statistics. The clustering of the outdoor temperatures with DTW-SOM revealed different patterns in warm and cool seasons. The identified diurnal temperature patterns in summer have higher values and more distinctive noon-time peaks than winter. Aghabozorgi et al.^5^ suggested time-series clustering can be improved by advancements in four different aspects: (1) dimension reduction; (2) clustering algorithms; (3) similarity measurements; and (4) prototypes; and concluded that future work should focus on new hybrid algorithms using existing or new clustering approaches in order to balance the quality and expense of clustering time-series. DTW-SOM introduced shape-based similarity measurement into the training phase of the standard SOM and improved the quality of clustering results on time series data. This new method can support time series clustering and pattern recognition.

## Data Availability

The datasets generated and analyzed during the current study are available from the corresponding author on reasonable request.
